# Grape Seed Extract Dose-Responsively Decreases Disease Severity in a Rat Model of Mucositis; Concomitantly Enhancing Chemotherapeutic Effectiveness in Colon Cancer Cells

**DOI:** 10.1371/journal.pone.0085184

**Published:** 2014-01-21

**Authors:** Ker Yeaw Cheah, Gordon Stanley Howarth, Susan Elaine Putnam Bastian

**Affiliations:** 1 School of Agriculture, Food and Wine, Waite Campus, University of Adelaide, South Australia, Australia; 2 Centre for Paediatric and Adolescent Gastroenterology, Children, Youth and Women's Health Service, North Adelaide, South Australia, Australia; 3 School of Animal and Veterinary Sciences, Roseworthy Campus, University of Adelaide, South Australia, Australia; University Hospital Carl Gustav Carus Dresden, Germany

## Abstract

**Objective:**

Mucositis is a serious disorder of the gastrointestinal tract that results from cancer chemotherapy. We investigated the effects of increasing grape seed extract doses on the severity of chemotherapy in a rat model and its coincident impact on chemotherapeutic effectiveness in colon cancer cells.

**Design:**

Female Dark Agouti rats were gavaged with grape seed extract (400–1000 mg/kg) or water (day 3–11) and were injected intraperitoneally with 5-Fluorouracil (150 mg/kg) or saline (control) on day 9 to induce mucositis. Daily metabolic data were collected and rats were sacrificed on day 12. Intestinal tissues were collected for histological and myeloperoxidase analyses. Caco-2 cell viability was examined in response to grape seed extract in combination with 5-Fluorouracil by 3-(4,5-Dimethylthiazol-2yl)-2,5-diphenyl-tetrazolium bromide) assay.

**Results:**

Compared with 5-Fluorouracil controls, grape seed extract (400–1000 mg/kg) significantly decreased the histological damage score (*P*<0.05) in the jejunum. Grape seed extract (1000 mg/kg) increased jejunal crypt depth by 25% (*P*<0.05) in 5-Fluorouracil treated rats compared to 5-Fluorouracil controls, and attenuated the 5-Fluorouracil -induced reduction of mucosal thickness (25%, *P*<0.05). Grape seed extract (600 mg/kg) decreased myeloperoxidase activity by 55% (*P*<0.01) compared to 5-Fluorouracil controls. Grape seed extract was more effective at ameliorating 5-Fluorouracil induced intestinal injury, with effects most pronounced in the proximal jejunum. Grape seed extract (10–25 ug/mL) significantly enhanced the growth-inhibitory effects of 5-Fluorouracil by 26% (*P*<0.05) in Caco-2 cells and was more potent than 5-Fluorouracil at 50–100 µg/mL.

**Conclusion:**

Grape seed extract may represent a new therapeutic option to decrease the symptoms of intestinal mucositis while concurrently impacting on the viability of colon cancer cells.

## Introduction

Mucositis is a serious, debilitating consequence of cancer therapy, which significantly reduces quality of life in cancer patients [1]. Mucositis is a painful condition associated with inflammation and ulceration of the gastrointestinal tract; most commonly affecting the mucosa of the mouth (oral mucositis) and small intestine (intestinal mucositis). Intestinal mucositis is characterized by reduced enterocyte proliferation and increased apoptotic rate of crypt cells, resulting in malabsorption and disrupted barrier function [2], [3]. Symptoms of mucositis include intense pain, diarrhoea, nausea, vomiting and anorexia. Often there is an increased risk of bacterial infection with associated mortality and morbidity [1]. Sometimes, gastrointestinal toxicity may lead to a reduction, or even termination, of the chemotherapy regimen [4]. Due to this, the chemotherapy dose administered to cancer patients often be sub-optimal; hence new regimens that reduce side-effects, maintaining efficacy are sought. Current mucositis treatments are largely ineffective as they target only the symptoms, but not the pathogenesis of the condition [5]. Thus, it is important to seek new alternative treatments which not only target mucositis but also enhance chemotherapeutic action without compromising the well-being of the patient. At present, the optimal combination of agents which could enhance both chemotherapeutic cytotoxicity against cancer cells and have minimal impact on normal cells, has not yet been determined.

Grape seed extract (GSE) is widely consumed as a dietary supplement on the basis of its potent anti-oxidant [6], anti-inflammatory [7] and purported, anti-neoplastic [8] properties. Procyanidins (PCs) are a class of polyphenolic compounds composed of flavan-3-ol subunits (oligomers and polymers) [9]. PCs are widely found in other food sources such as tea, apples and red wine and are believed to be the key bioactive constituents in GSE [10], [11]. Several studies have reported that the absorption and bioavailability of PCs in the gut is dependent upon their chemical structure and degree of polymerization [12]. PCs (degree of polymerization = 7 or higher) are retained in the intestinal tract, thereby increasing contact time with gut enterocytes to promote intestinal health [10]. Studies examining GSE in combination with 5-Fluorouracil (5-FU) in normal animals are limited. In addition, no studies have been published examining the combination of 5-FU and GSE in colon cancer models.

Previously, we demonstrated in a preliminary study that GSE (400 mg/kg) was able to reduce intestinal damage both in rat models of intestinal mucositis [13] and ulcerative colitis [14]. However, the dose required to achieve maximal therapeutic benefit, dose-responsiveness and safety of GSE remained undefined. Accordingly, we investigated GSE across a range of doses for its potential to optimally and safely reduce the severity of intestinal mucositis in a rat model. Increasing doses of GSE further prevented 5-FU-induced mucositis damage, and these treatments were well tolerated by the animals as no metabolic changes were observed compared to the healthy controls. In addition, we also investigated the effects of the combination of GSE with 5-FU chemotherapy on colonic neoplasia in an *in vitro* model compared to the effectiveness of 5-FU alone. The combination of GSE and 5-FU further enhanced toxicity in colon cancer cells.

## Materials and Methods

### Chemicals

Catechin, epicatechin, methanol, phloroglucinols, ascorbic acid hexadecyltrimethylammonium bromide (HTAB), sodium bicarbonate and *o*-dianisidine were purchased from Sigma Chemical Co. Ltd, St Louis, MO. Folin-ciocalteau reagent and ^13^C sucrose were purchased from AnalaR, BDH, MERCK, Pty. Ltd., Australia. Tissue culture solutions include Dulbecco's Modified Eagle's Minimum Essential Medium (DMEM), fetal calf serum (FCS), antibiotics (penicillin, gentamicin and streptomycin), Dulbecco's Phosphate Buffered Saline, dimethyl sulfoxide (DMSO) and 3-(4,5-Dimethylthiazol-2yl)-2,5-diphenyl-tetrazolium bromide) (MTT) were purchased from Gibco BRL, Life Tehnologies Pty Ltd, Australia. The chemotherapy drugs, 5-Fluorouracil (5-FU) were purchased from MaynePharma Pty. Ltd., Australia).

### Grape seed extract (GSE) preparation

Grape seed extract was kindly donated by Tarac Techologies (North Adelaide, South Australia, batch no: 02VIN03) and stored in an air-tight, light-resistant pack until being dissolved in distilled water prior to use. The GSE was derived from condensed tannin made from Australian white wine marc (residual skins and seeds from winemaking). The nutrition profile of GSE is listed in Table 1. The GSE utilized in the current study was obtained from the same source with the same batch number to that described by Cheah et al [13]. The polyphenolic content was measured previously by Folin-ciocalteau assay [13], and the chemical profile was quantified by phloroglucinolysis described below.

**Table 1:** Nutritional and phenolic content of grape seed extract (GSE).

Nutrition profile^1^	Qty per 100 g
Energy (KJ)	1480
Protein (g)	4.5
Fat (g)	0.2
Carbohydrate (g)	
total	79.1
sugars	0.2
Sodium (g)	0.3
**Phenolic profile** 2	
Polyphenol^6^ (%)	43

1 The nutrition profile of GSE is represented as quantity/100 g.

2 The phenolic content was obtained from Folin-ciocalteau (FC) assays.

### Folin-ciocalteau assay

The total phenolic content of GSE was previously quantified by Folin-ciocalteau assay [13]. Briefly, GSE samples were added in triplicate to non-sterile 96 well plates and incubated with Folin-ciocalteu reagent for 5 min. Sodium bicarbonate solution (7.5% w/v) was added and further incubated for 4 h in the dark. The plate was read at 740 nm by a spectrometer (Multiskan® Spectrum, Therma Electron Corporation, Vantaa, Finland) using Skanit software 2.2. Catechin standards were prepared from 1 mg/mL stock (1/2 serial dilutions) and used to generate a calibration curve. Data were analysed using GraphPad Prism version 4.0 for windows® (GraphPad Software, San Diego, CA, USA) and expressed in mg/mL catechin equivalents.

### Quantification of procyanidins (PCs) in GSE by phloroglucinolysis

The procyanidin profile of GSE was characterized by phloroglucinolysis which determines the subunit composition, mean degree of polymerization (mDP) and galloylation of PCs. Phloroglucinolysis was performed according to a previously described method [15]. GSE was dissolved in methanol (10 mg/mL, v/v) and 25 µL of GSE was added to an equal volume of phloroglucinol solution (0.2 N HCL in methanol, 100 g/L of phloroglucinol and 20 g/L of ascorbic acid). The phloroglucinolysis reaction was carried out at 50°C for 25 min and analyzed by reverse phase-high pressure liquid chromatography (RP-HPLC) using (−)-epicatechin as quantitative standard [15].

### Ethic Statements

This study followed the Australian Code of Practice for the Care and Use of Animals for Scientific Purposes and was approved by both the Animal Care and Ethics Committees of the Children, Youth and Women's Health Service and the University of Adelaide (AE:777-3-2011).

### Animal studies

Female Dark Agouti rats (100–140 g, n = 64) were housed in individual metabolic cages (Tecniplast, Exton, PA, USA) in a temperature-controlled room (22°C) with a light-dark cycle of 12 h. Rats were given *ad libitum* access to water and food (18% casein-based diet) [16] in the Animal Care Facility of the Children, Youth and Women's Health Service, North Adelaide, South Australia.

Rats were randomly allocated to 8 groups (n = 8): Water+Saline injection; GSE 400 mg/kg+Saline injection; GSE 600 mg/kg+Saline injection; GSE 1000 mg/kg+Saline injection; Water+5-FU injection (5-Fluorouracil: 150 mg/kg); GSE 400 mg/kg+5-FU injection; GSE 600 mg/kg+5-FU injection; and GSE 1000 mg/kg+5-FU injection. Rats were acclimatized in metabolism cages from day 0–2 and then gavaged with 1 mL GSE dissolved in water (400 mg/kg, 600 mg/kg or 1000 mg/kg) or water from day 3–11. At day 9, all rats were intraperitoneally injected with either 5-FU or saline (controls). Daily measurements of body weight, food and water intake, and urine and faecal output were recorded. Rats were sacrificed by CO_2_ asphyxiation followed by cervical dislocation on day 12. All visceral organs were weighed and discarded. The lengths and weights of the gastrointestinal organs (duodenum, small intestine and colon) were recorded. Representative samples (2 cm) of gastrointestinal organs were collected and fixed in 10% buffered formalin for histological analyses, while four cm samples were snap frozen in liquid nitrogen and stored at −80°C for biochemical analysis.

### ^13^C-sucrose breath test (SBT)

The SBT is an indirect measure of intestinal sucrase activity and was performed according to the method described by Tooley *et al.* [17] In brief, rats were oro-gastrically gavaged with 1 mL of sucrose solution containing ^13^C (250 mg/kg) and breath samples were collected at 15 min intervals for 120 min. Breath samples were analyzed for ^13^CO_2_ content by isotope ratio mass spectrometry (IRMS) equipped with a V410 data collection system (Europa Scientific, ABCA 20/20, Crewe, United Kingdom). The data were expressed as percentage cumulative dose at 90 min (%CD90) by calculating the change in breath ^13^CO_2_ levels from baseline for each time point of breath collection throughout the period of interval sampling. SBT determinations were performed at day 3 (before GSE treatment), day 9 (before 5-FU injection) and day 12 (before kill).

### Myeloperoxidase (MPO) assay

Small intestinal tissue samples (4 cm) of jejunum, junction of jejunum and ileum (JI) and ileum were thawed on ice and homogenized with 1.5 mL of phosphate buffer (10 mM, pH 6.1) for 60 seconds until the solution was homogenous. The homogenates were kept frozen at −80°C until required.

MPO is an enzyme present in the intracellular granules of neutrophils, acting as an acute inflammation marker. The level of MPO in the small intestine was determined by a slight modification of the assay described by Krawisz *et al.* [18] Tissue homogenates were thawed on ice and centrifuged at 13000 g for 13 min. The supernatant was discarded and cell pellets were re-suspended in hexadecyltrimethyl ammonium bromide (0.5%, pH 6.0). The samples were vortexed for 2 min and further centrifuged at 13000 g for 3 min. Supernatants were reacted with *o*-dianisidine and absorbance measured at 450 nm at 1 min intervals for a period of 15 min using a microplate reader (Sunrise Microplate Reader, Tecan Austria GmbH, Grodig, Austria). MPO activity was expressed as units MPO activity per gram of tissue.

### Histological analyses

Gut tissue samples (2 cm) were embedded in paraffin wax and 4 µm sections were stained with haemotoxylin and eosin. The overall histological disease severity score (ODS) of intestinal sections was rated semi-quantitatively (0–3) based on 11 independent histological criteria according to a protocol described by Howarth *et al.* [19] Villus heights and crypt depths (40 villi and 40 crypts per section) were determined in the small intestinal sections including jejunum, junction of jejunum and ileum (JI) and ileum as described in Howarth *et al.* [19] The combined measurement of villus heights and crypt depths provided an approximation of total mucosal thickness in each small intestinal specimen. All microscope-based analyses were performed in a blinded fashion using a light microscope (Nikon, ProgRes®CS, Tokyo, Japan) and image ProPlus software version 5.1 (Media Cybernetics, Silver Spring MD, USA).

### Cell Culture

The human colon cancer cell line, Caco-2 was obtained from the American Type Culture Collection (ATCC, Manassas, USA). Caco-2 cells were maintained at 37°C in a humidified incubator with 5% CO_2_ – 95% air, and 90% relative humidity in Dulbecco's Modified Eagle's Minimum Essential Medium (DMEM) supplemented with 10% (v/v) fetal calf serum (FCS) and 1% antibiotics (penicillin, gentamicin and streptomycin) (v/v). The cells were grown in 75 cm^2^ vented tissue culture flasks, culture medium was changed twice a week and cells were passaged when they were 80–90% confluent.

### Cell viability

The inhibition of Caco-2 cells viability was determined by 3-(4,5-Dimethylthiazol-2yl)-2,5-diphenyl-tetrazolium bromide) (MTT) assay according to a previously described method by Huynh-Delerme *et al.* [20] Cells (5×10^3^ cells/well) were seeded on 96-well tissue culture plates for 48 hrs to allow attachment. GSE was prepared in dimethyl sulfoxide (DMSO) and diluted with DMEM and further filter-sterilized through a 0.22 µM filter (Millipore, South Australia, Australia). In all experiments, the concentration of DMSO in control and treated samples was less than 0.025%. After 48 h, culture medium was replaced with serum free media containing GSE at different concentrations (µg/mL) and 5-FU (µM) and further incubated for either 24 or 48 h. MTT solution was prepared in Dulbecco's Phosphate Buffered Saline (1 mg/mL) and sterile-filtered to remove any biological contaminants. Next, 50 µL of MTT solution was added to each well and further incubated at 37°C for 4 h. Medium was replaced with 100 µL of DMSO to extract the formazan product. Plates were placed on a shaking incubator for 15 min and read by spectrometer at 570 nm. Data were expressed as number of viable cells as a percentage of control cells treated with serum free medium only.

### Statistical analyses

Statistical analyses were conducted using PASW 18 (SPSS, Inc., Chicago, IL, USA) and XLSTAT version 2011.4.02 (Addinsoft SARL, France). All parametric data including bodyweight, daily metabolic data, SBT, MPO, villus height and crypt depth and cell viability were compared using one-way analysis of variance (ANOVA) with a Tukey's *post-hoc* test. The overall disease severity score (ODS) was compared by a Kruskal-Wallis test with a Mann Whitney U-test to identify significance between groups. Data were considered significant at *P*<0.05.

## Results

### Characterization of procyanidins by phloroglucinolysis

GSE had low mass conversion (23.8% w/w), mDP (5.9) and molecular mass (1871 g/mol), as measured by phloroglucinolysis (Table 2). The PC terminal subunits in GSE were mostly dominated by (−)-epicatechin-3-*O*-gallate.

**Table 2:** The chemical profiles of grape seed extract characterized by phloroglucinolysis.

	MC^1^	mDP^2^	galloylation	MM^3^	Terminal subunits^4^		Extension subunits^4^			
	(%)		(%)	(subunit)	C	E	ECG	C-P	E-P	ECG-P
GSE	23.8	5.9	19	1871	20.1	14.0	65.9	8.9	53.0	38.1

1 Mass conversion based on % recovery of procyanidin by phloroglucinolysis based on the gravimetric mass.

2 Mean degree of polymerization.

3 Estimated molecular mass based on subunit composition from phloroglucinolysis.

4 Percent composition of terminal and extension subunits (in moles) with the following subunit abbreviations: (-P), phloroglucinol adduct of extension subunit; C, (+)-catechin; EC, (−)-epicatechin; ECG, (−)-epicatechin-3-*O*-gallate.

### Daily metabolic parameters and bodyweight

Oral administration of GSE (400, 600 or 1000 mg/kg) between days 3 and 9 did not significantly affect body weight, food or water intake and urine or faecal output compared to rats receiving water (Table 3). 5-FU injection significantly increased water intake and urine output and reduced body weight, food intake and faecal output compared to rats receiving saline injection between days 10 and 12 (Table 4). GSE 600 and 1000 mg/kg in 5-FU treated rats significantly returned faecal output (*P*<0.01), back towards the values for normal saline-injected control rats.

**Table 3:** Effects of increasing doses of grape seed extract (GSE; mg/kg) on cumulative body weight change, food and water intake, urine and faecal output in saline-injected rats over day 3–9.

	Day 3–9			
	Water	GSE 400	GSE 600	GSE 1000
	(n = 16)	(n = 16)	(n = 16)	(n = 16)
**Body Weight Change (g)**	12.0±0.9	11.7±0.7	10.5±1.3	11.1±0.8
**Water Intake (mL)**	174.2±7.9	175.5±6.8	171.6±7.9	185.1±10.1
**Food Intake (g)**	66.2±0.8	63.8±1.1	62.4±1.6	62.2±0.9
**Urine Output (mL)**	117.8±6.7	115.2±4.9	112.7±5.9	127.8±8.4
**Faecal Output (g)**	8.2±0.1	8.5±0.3	8.8±0.3	9.1±0.3

Data are expressed as means ± SEM.

**Table 4:** Effects of increasing doses of grape seed extract (GSE; mg/kg) on cumulative body weight change, food and water intake, urine and faecal output in saline-injected rats and in 5-Fluorouracil (5-FU) injected rats from day 10–12.

	Day 10–12				
	Water+Saline	Water+5-FU	GSE 400+5-FU	GSE 600+5-FU	GSE 1000+5-FU
	(n = 8)	(n = 8)	(n = 8)	(n = 8)	(n = 8)
**Body Weight Change (g)**	−4.3±1.1	−8.9±0.8*	−9.8±0.5**	−9.4±0.9**	−9.8±0.1**
**Water Intake (mL)**	73.3±2.0	109.4±10.8*	100.0±5.3	89.8±12.2	113.4±10.7*
**Food Intake (g)**	28.7±0.8	13.8±0.9***	13.8±0.8***	12.7±1.4***	13.3±1.0***
**Urine Output (mL)**	56.3±2.6	85.8±6.4*	87.3±4.5*	80.0±7.0*	89.8±11.0*
**Faecal Output (g)**	3.6±0.2	2.3±0.3**	2.8±0.2	3.5±0.3##	3.3±0.1#

Data are expressed as means ± SEM. Statistical significance compared to water+saline, where

* indicates *P*<0.05,

** *P*<0.01 and

*** *P*<0.001,

# *P*<0.05 and

## *P*<0.01 compared to Water+5-FU.

### Visceral organs

5-FU injection significantly reduced thymus weight by 51% (*P*<0.001) and spleen weight by 20% (*P*<0.01) compared to saline-injected rats. Whilst GSE did not prevent any of the 5-FU-induced changes in thymus and spleen weight, none of the GSE doses tested impacted negatively on visceral organ weights (Table 5) nor gastrointestinal organ weights and lengths (Table 6) in healthy animals. However, GSE 1000 mg/kg significantly increased stomach weight by 13% (*P*<0.05) compared to normal controls.

**Table 5:** Effect of increasing doses of grape seed extract (GSE; mg/kg) on organ weights of female Dark Agouti rats 72 h after 5-Fluorouracil (5-FU) or saline injection.

	Water+Saline	GSE 400+saline	GSE 600+Saline	GSE 1000+Saline	Water+5-FU	GSE 400+5-FU	GSE 600+5-FU	GSE 1000+5-FU
	(n = 8)	(n = 8)	(n = 8)	(n = 8)	(n = 8)	(n = 8)	(n = 8)	(n = 8)
**Heart**	397±6	393±6	406±8	402±8	405±7	414±16	408±6	403±6
**Lung**	611±8	593±10	625±18	657±38	721±49	711±40	663±47	580±58
**Liver**	3111±57	3008±43	3072±63	3114±45	3370±144	3334±85	3353±73	3311±41
**Kidneys**	832±26	816±15	815±18	857±13	874±20	896±21	880±13	891±19
**Thymus**	181±18	148±17	178±13	183±14	88±18***	86±7***	91±15***	69±8***
**Spleen**	198±5	205±5	193±8	205±4	159±6**	155±5**	150±13***	153±3***
**Stomach**	600±10	630±10	640±10	680±10*	590±30	650±10	650±20	680±20*
**Caecum**	370±10	360±10	410±40	400±10	470±40	490±50	440±30	440±30

Organ weights are expressed as (wt g/kg bwt) %. Data are expressed as means ± SEM. Statistical significance compared to Water+Saline,

* indicates *P*<0.05,

** *P*<0.01 and

*** *P*<0.001.

**Table 6:** Effects of increasing grape seed extract (GSE; mg/kg) doses on gastrointestinal organ weights and lengths of female Dark Agouti rats 72 h after 5-Fluorouracil (5-FU) or saline injection.

	Water+Saline	GSE 400+saline	GSE 600+Saline	GSE 1000+Saline	Water+5-FU	GSE 400+5-FU	GSE 600+5-FU	GSE 1000+5-FU
	(n = 8)	(n = 8)	(n = 8)	(n = 8)	(n = 8)	(n = 8)	(n = 8)	(n = 8)
**Duodenum**								
Weight (g/kg)	20±1	18±1	20±1	22±1	21±1	21±1	20±1	24±2
Length (cm)	506±28	540±30	520±20	560±10	530±10	520±20	520±20	530±30
**Jejunum+Ileum**								
Weight (g/kg)	199±5	213±5	204±5	228±5	193±3	193±5	198±7	2080±4
Length (cm)	7100±140	7250±110	7040±160	7380±190	6850±110	6840±90	7090±120	6860±60
**Colon**								
Weight (g/kg)	53±2	53±2	52±2	56±2	64±3	55±2	63±6	60±4
Length (cm)	1180±40	1130±40	1090±50	1210±50	1020±40	1050±40	1140±40	1150±20

Gastrointestinal organ weights are expressed in (wt g/kg bwt)×100% and lengths are expressed in (cm). Data are expressed as means ± SEM.

### Sucrose breath test (SBT)

**Figure 1:**
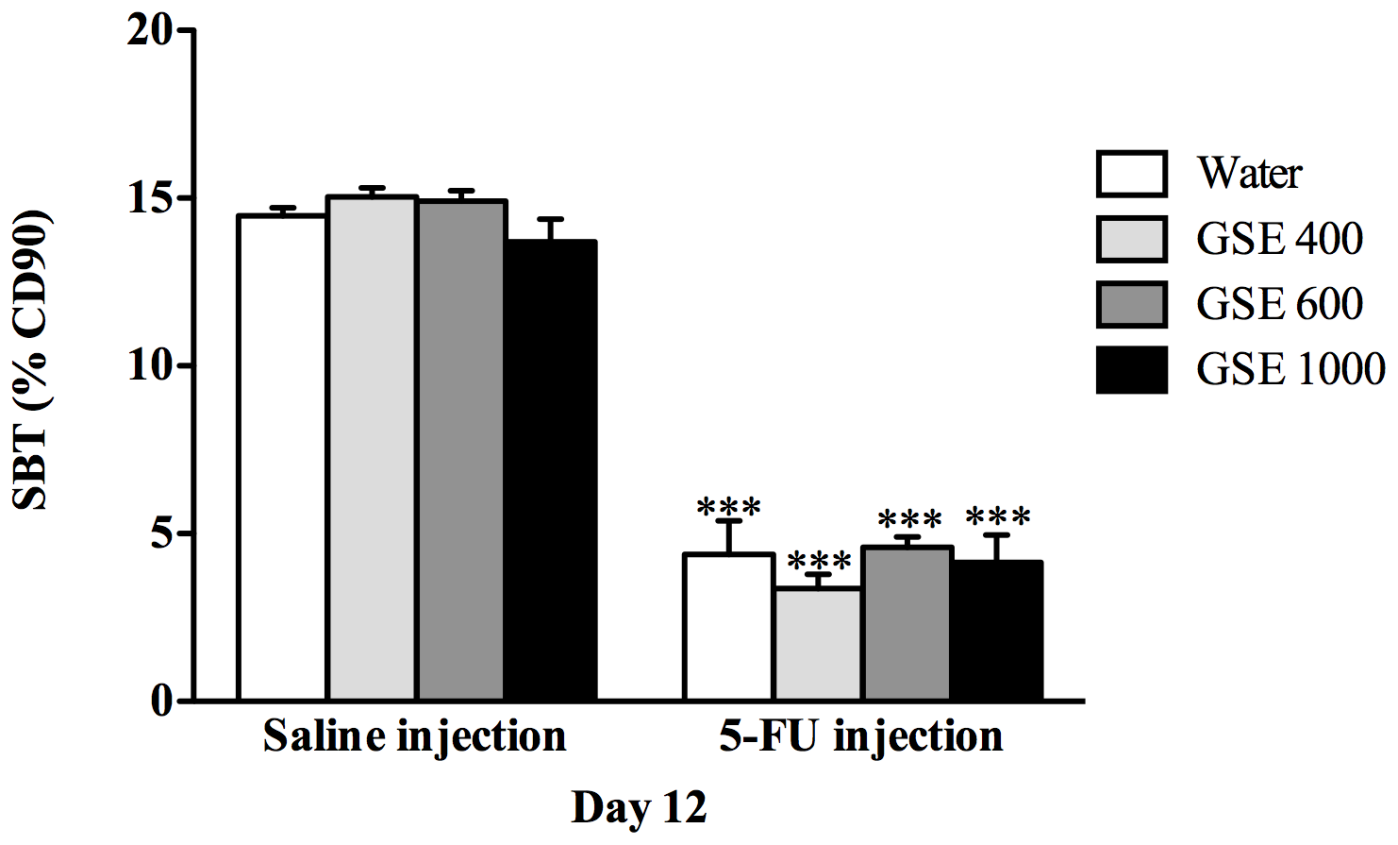
Effects of GSE (mg/kg) on small intestinal sucrase activity assessed by the sucrose breath test on day 12 (72 h after 5-FU or Saline injection). Data expressed as mean (%CD90) ± SEM. *** indicates *P*<0.001 compared to Water+Saline.

5-FU injection significantly (*P*<0.001) decreased the SBT (%CD90) by 70% compared to values in water+saline treated rats, indirectly indicating 5-FU injection had disrupted brush border sucrase activity (Figure 1). There were no significant differences in %CD90 among any of the 5-FU treated rats receiving GSE compared to 5-FU treated-control rats. In addition, no significant differences in %CD90 were observed between GSE and water treatment, implying none of the GSE doses had demonstrably impacted on brush border sucrase activity in healthy rats (Figure 1).

### Myeloperoxidase activity (MPO)

**Figure 2:**
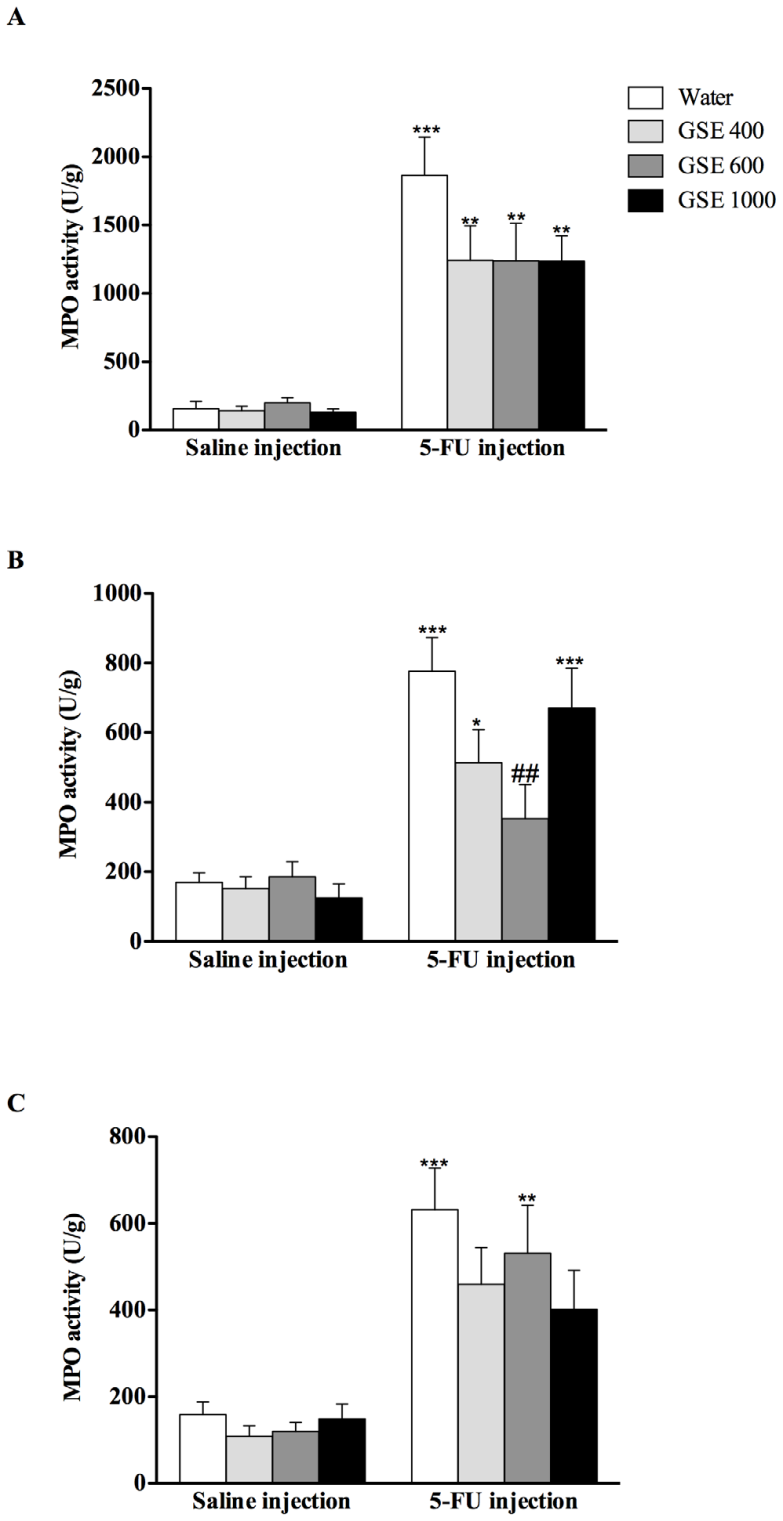
Effects of GSE (mg/kg) on Myeloperoxidase (MPO) activity in the jejunum (A), JI (B) and ileum (C) 72 h after either saline or 5-FU injection. Data are expressed as mean (MPO units/g tissue) ± SEM. * indicates *P*<0.05, ** indicates *P*<0.01 and *** indicates *P*<0.001 compared to rats receiving water and saline injection. ## indicates *P*<0.01 compared to rats receiving water and 5-FU injection.

Following 5-FU injection, there was a significant (*P*<0.001) increase in MPO activity in the proximal jejunum, junction of jejunum and ileum (JI) and ileum of water+5-FU treated rats (1092%, 357% and 297% respectively) compared to water+saline treatment (Figure 2). GSE 600 mg/kg significantly (*P*<0.01) reduced MPO activity by 55% in the JI compared to water+5-FU treated rats (Figure 2B). There was no significant difference in MPO activity between GSE and water treatment in healthy rats (Figure 2), indicating that GSE administration did not affect MPO activity in healthy animals.

### Overall disease severity scores

**Figure 3:**
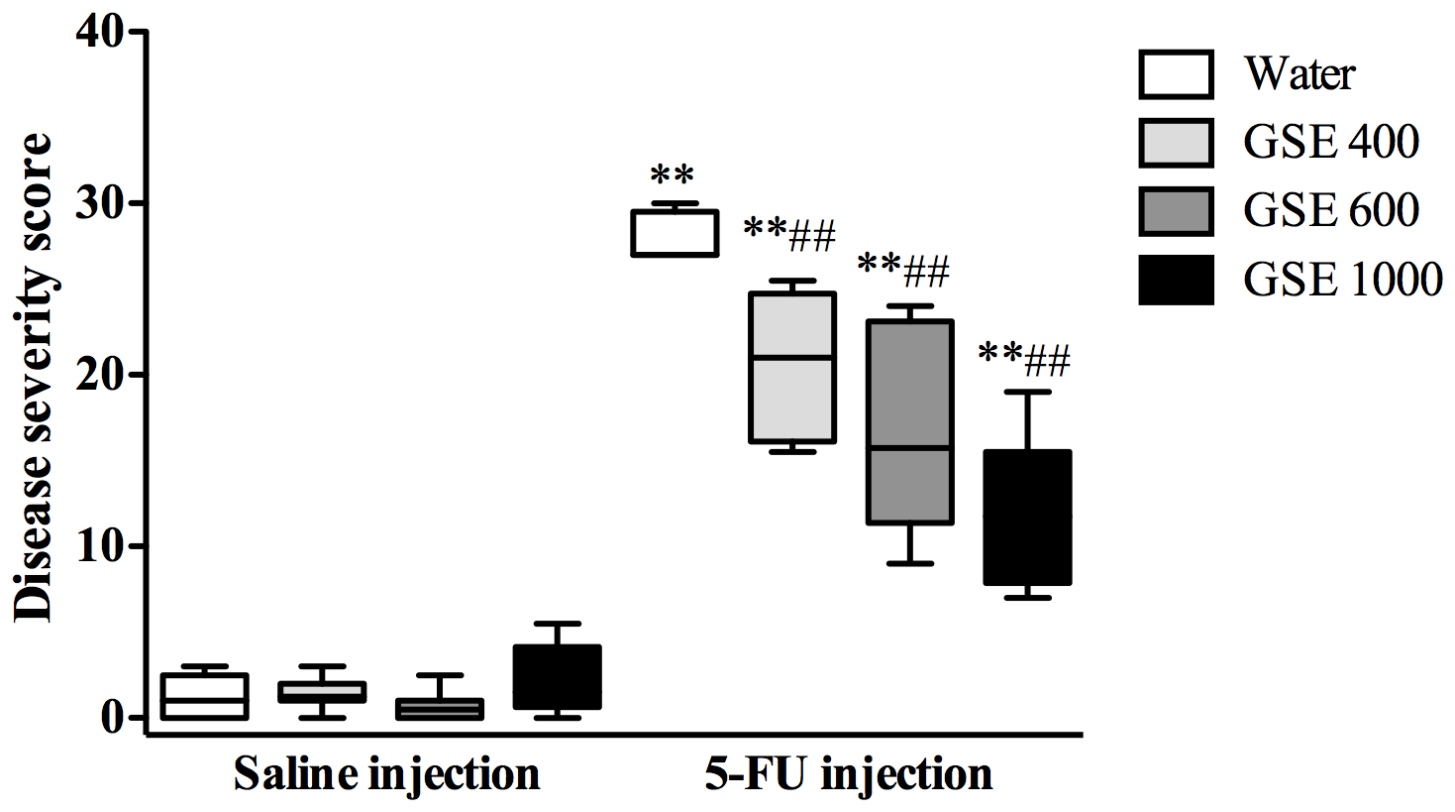
Effects of GSE (mg/kg) on histological severity scores in the jejunum 72 h after either saline or 5-FU injection. The severity scores were rated based on 11 parameters on different layers of intestinal tissues based on previously described protocol Howarth *et al.* [19] The box plots represent the range of disease severity score and the horizontal lines represent the median disease severity score. ** indicates *P*<0.01 compared to Water+Saline. ## indicates *P*<0.01 compared to Water+5-FU.

**Figure 4:**
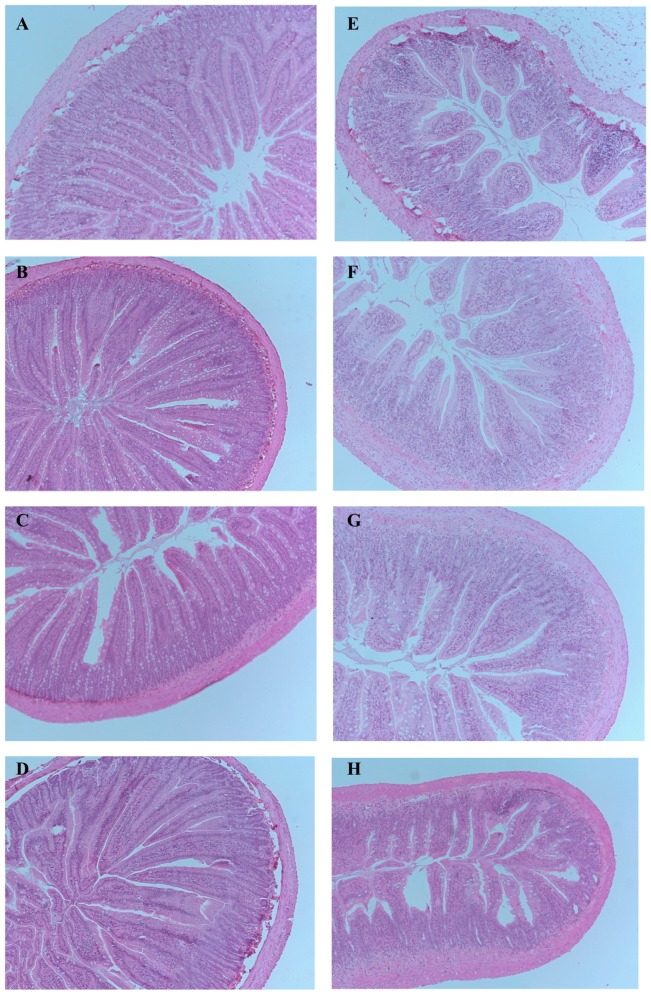
Representative photomicrographs of the proximal jejunum sections stained with haematoxylin and eosin in Water+Saline (A), GSE 400 mg/kg+Saline (B), GSE 600 mg/kg+Saline (C), GSE 1000 mg/kg+Saline (D), Water+5-FU (E), GSE 400 mg/kg+5-FU (F), GSE 600 mg/kg+5-FU (G) and GSE 1000 mg/kg+5-FU (H). (Original magnification 40×.)

Administration of 5-FU significantly increased disease severity score in the proximal jejunum when assessed by the semi-quantitative histological severity score analysis (Figure 3 and 4). 5-FU controls attained the highest damage score (median score = 30) and were significantly greater than water+saline treated rats (median score = 1, *P*<0.01). GSE treatment significantly reduced disease severity score in 5-FU treated rats in a dose-responsive manner (GSE 400 = 21 (15.5–25.5), *P*<0.01; GSE 600 = 15.75 (9–24), *P*<0.01; and GSE 1000 = 11.75 (7–19), *P*<0.01) compared to 5-FU controls. No significant difference in disease severity score was observed between GSE and water treated rats receiving saline injection (Figure 3), indicating that GSE had not disrupted intestinal integrity in healthy animals.

### Villus height, crypt depth and mucosal thickness

**Figure 5:**
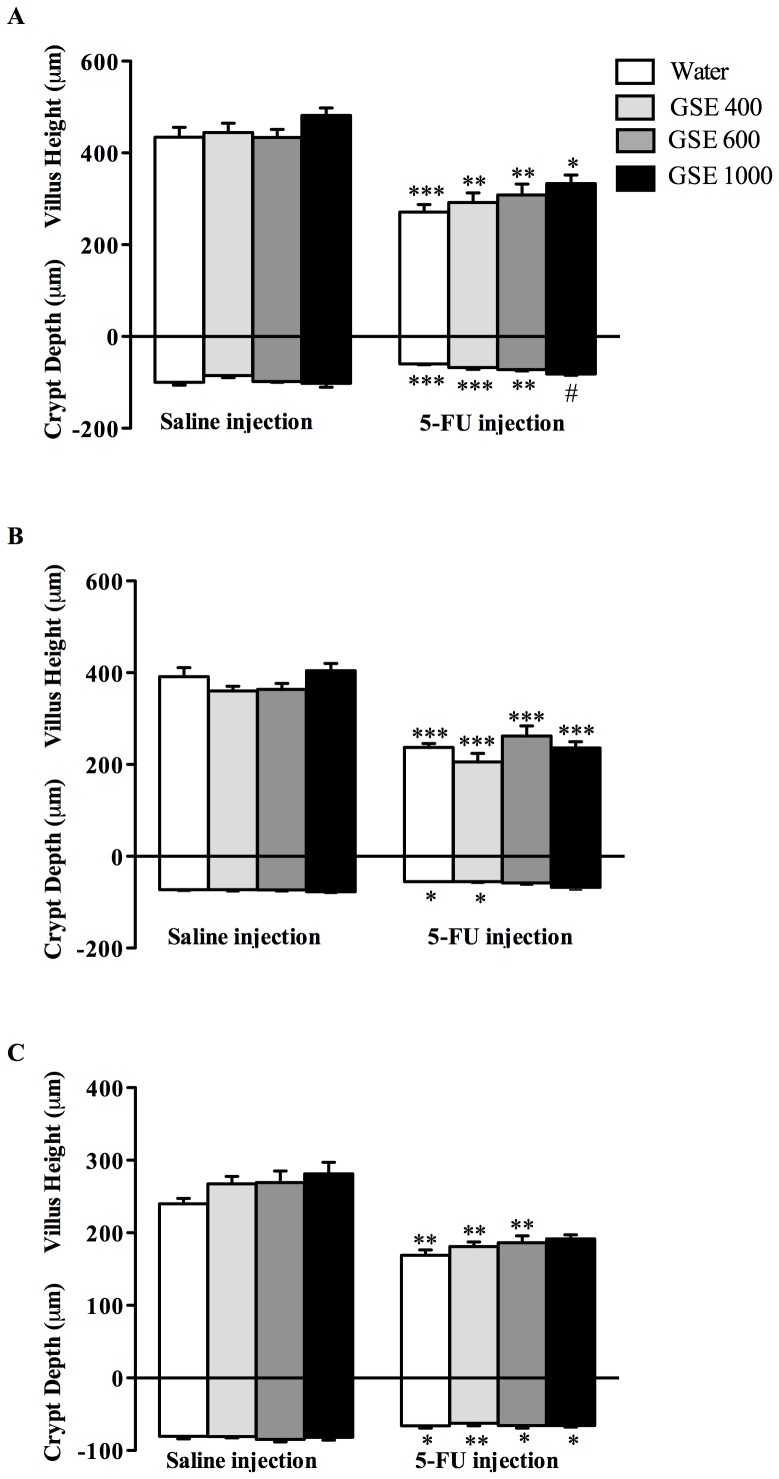
Effects of GSE (mg/kg) on villus height and crypt depth in the jejunum (A), JI (B) and Ileum (C) 72 h after either saline or 5-FU injection. Data are expressed as mean (µm) ± SEM. * indicates *P*<0.05, ** indicates *P*<0.01 and *** indicates *P*<0.001 compare to Water+Saline. # indicates *P*<0.05 compared to Water+5-FU.

**Figure 6:**
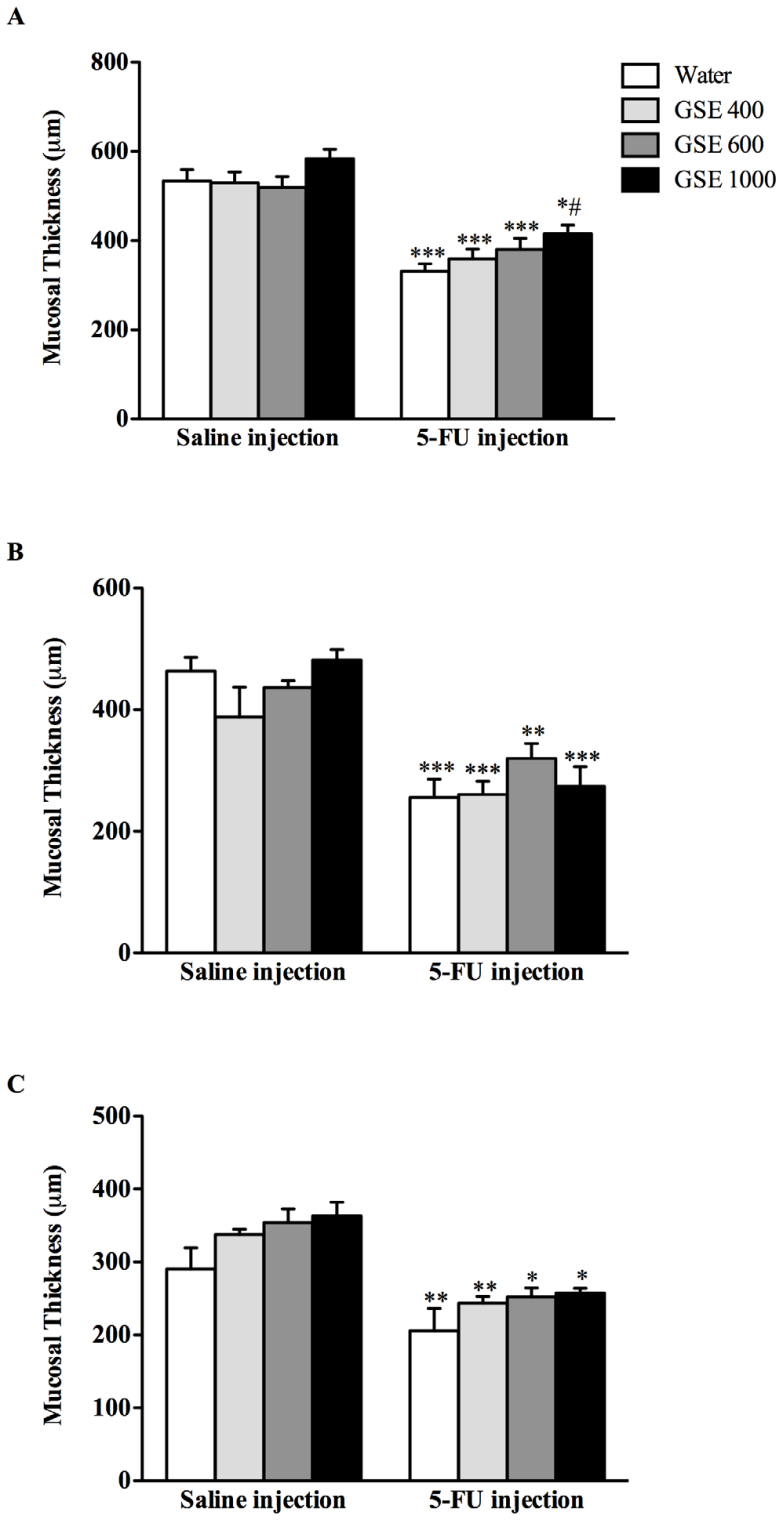
Effects of GSE (mg/kg) on mucosal thickness in the jejunum(A), JI (B) and Ileum (C) 72 h after either saline or 5-FU injection. Data are expressed as mean (µm) ± SEM. * indicates *P*<0.05, ** indicates *P*<0.01 and *** indicates *P*<0.001. # indicates *P*<0.05 compared to Water+5-FU.

5-FU injection resulted in shortening of the villi in the jejunum (38%, *P*<0.001), JI (39%, *P*<0.001) and ileum (26%, *P*<0.01) compared to water controls (Figure 5). 5-FU injection also reduced crypt depth in the jejunum (38%, *P*<0.001), JI (23%, *P*<0.05) and ileum (15%, *P*<0.05). In the jejunum, GSE treatments tended to dose-responsively improve villus height and crypt depth, although only GSE at a dose of 1000 mg/kg significantly (*P*<0.05) increased crypt depth compared to 5-FU controls (Figure 5A). Importantly, none of the GSE treatments impacted negatively on villus height and crypt depth in healthy animals. 5-FU injection significantly reduced mucosal thickness in the jejunum (38%, *P*<0.001), JI (45%, *P*<0.001) and ileum (29%, *P*<0.01) compared to water controls (Figure 6). GSE treatments tended to dose-responsively increase mucosal thickness in the jejunum, although only GSE 1000 mg/kg significantly increased mucosal thickness (25%, *P*<0.05) compared to 5-FU controls (Figure 6A).

### Effects of 5-FU on viability of Caco-2 cells

**Figure 7:**
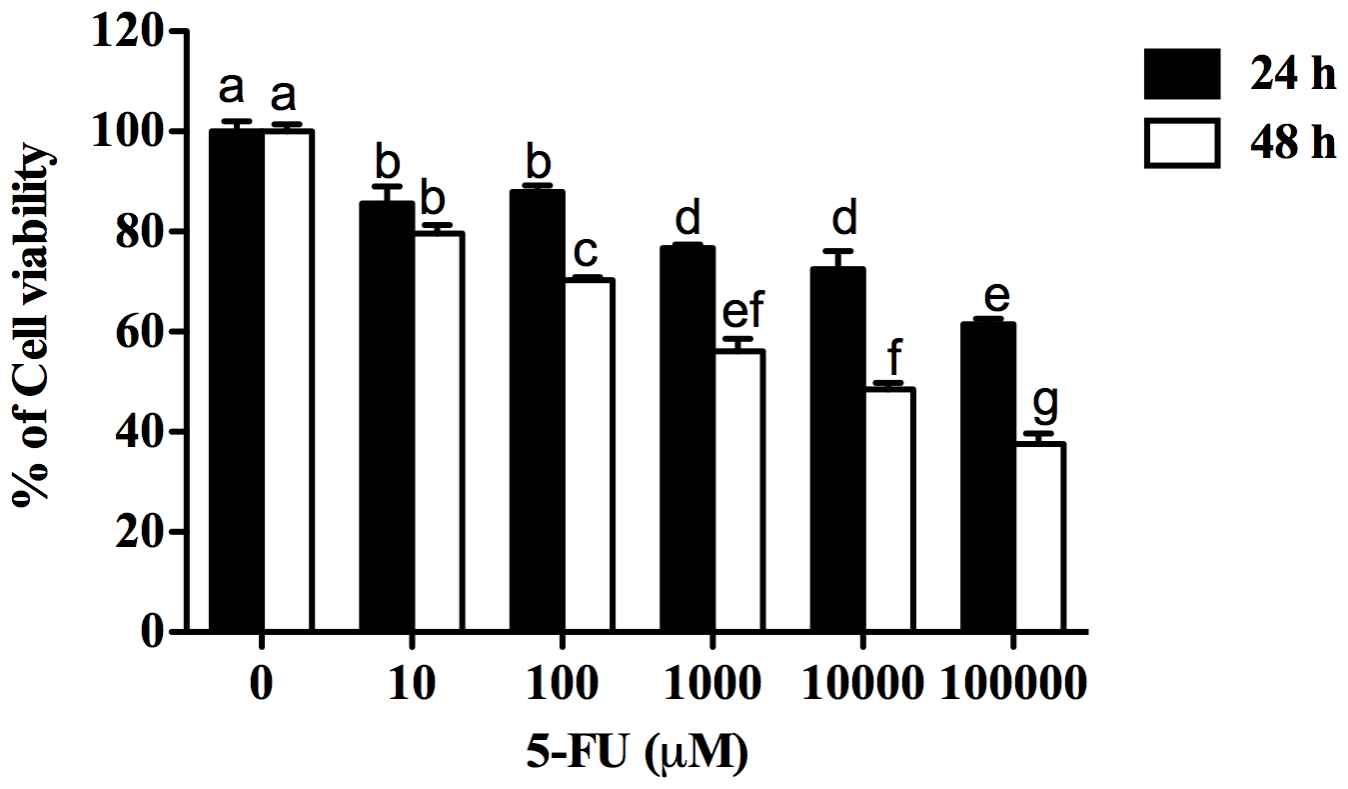
Viability of Caco-2 cells treated with 5-FU for either 24 h or 48 h, assessed by MTT assay. Data are expressed as percent of cell viability relative to viability of untreated controls. Data are presented as means ± SEM of 2–3 independent experiments. Bar data not sharing the same letter are significantly different *P*<0.05.

The dose responses of 5-FU (0–100,000 µM) on Caco-2 cells for 24 h and 48 h are illustrated in Figure 7. 5-FU doses were also tested at 72 h (Data not shown). The cell viability of Caco-2 cells was inhibited by 5-FU in a time- and dose-dependent manner. At 24 h, 5-FU at 100 µM significantly reduced viability in Caco-2 cells to 88% (*P*<0.05) of control values and a further reduction of cell viability was observed to 70% (*P*<0.05) of control values at 48 h. A 100 µM concentration of 5-FU was selected for the next experiment because this dose was able to reduce Caco-2 cell viability (70–85%) reflecting gastrointestinal toxicity commonly observed in cancer patients following chemotherapy.

### Effects of GSE and 5-FU on Caco-2 cell viability

**Figure 8:**
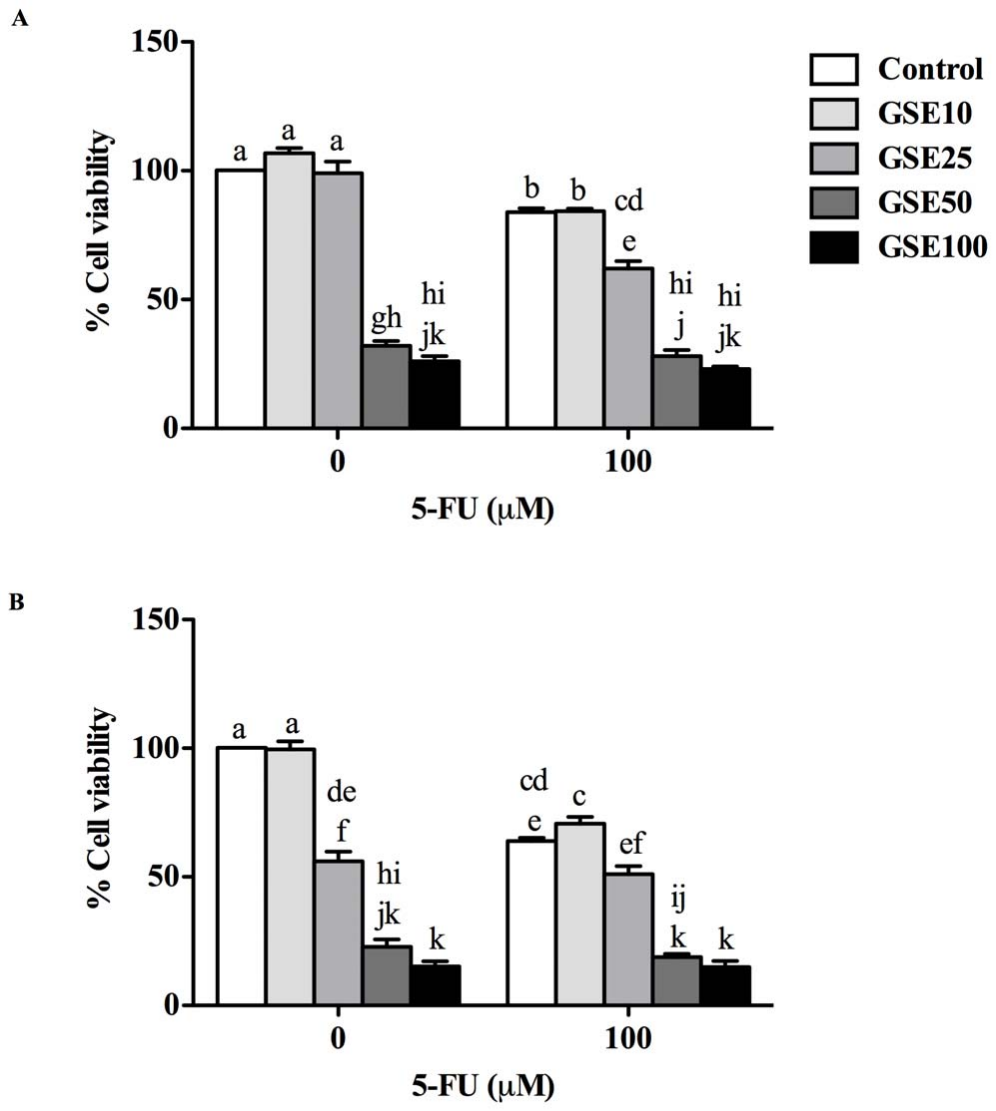
Viability of Caco-2 cells either treated with GSE alone (µg/mL) or GSE (µg/mL)+5-FU (100 µM) for either 24 h (A) or 48 h (B). Data are expressed as percent of cell viability relative to untreated controls. Data are presented as means ± SEM of 4 independent experiments. Bar data not sharing the same letters are significantly different *P*<0.05.

In order to establish the cytotoxicity of GSE on Caco-2 cells, GSE (10–100 µg/mL) was applied to cells for either 24 or 48 h (Figure 8). GSE treatment inhibited cell viability in a dose- and time-dependent manner. GSE treatments significantly (*P*<0.05) reduced cell viability (IC_50_ = 50.24 µg/mL) at 24 h and became more toxic to the Caco-2 cells at 48 h (IC_50_ = 37.84 µg/mL). When the cells were exposed to the combination of GSE (10–100 µg/mL) and 5-FU (100 µM), greater numbers of dead cells were evident compared to cells exposed to 5-FU alone (Figure 8). At 24 h, 5-FU significantly reduced cell viability to 84% (*P*<0.05) of control values. Interestingly, when Caco-2 cells were exposed to the combination of GSE and 5-FU, the growth inhibitory effects of 5-FU were significantly enhanced by 26% (GSE = 25 µg/mL; *P*<0.05; combined treatment vs. both agents alone) at 24 h. GSE at higher doses (50–100 µg/mL) exerted greater growth inhibition compared to 5-FU alone. At 24 h, GSE induced significant growth inhibitory effects on Caco-2 cells (GSE 50 = 33% and GSE 100 = 27%; *P*<0.05) compared to 5-FU control (84% of control value) (Figure 8A). In addition, GSE alone significantly (*P*<0.05) decreased the viability of Caco-2 cells (GSE 50 = 31% and GSE 100 = 29%; *P*<0.05) compared to 5-FU control (64% of control value) at 48 h (Figure 8B).

## Discussion

The present study represents the first report of GSE dose-responsively reducing severity of mucositis. Our findings suggest that higher doses of GSE are more effective at reducing the severity indicators of intestinal mucositis in rats and that these GSE-induced effects are largely dose-dependent and more evident in the proximal jejunum compared to the distal small intestine.

Injection of 5-FU impacts on the small intestine to a greater extent than the large intestine, presumably due to the greater cell turnover rate in the more proximal regions of the gut [3]. Our results are in agreement with previous studies [18], [19], [21], in which the 5-FU mucositis model resulted in severe intestinal injury 72 h after the induction of mucositis. This damage was characterized by a reduction of intestinal brush border enzyme activities [21], increased neutrophil infiltration [22], increased disease severity score [23] and decreased mucosal thickness [13]. Consistent with earlier studies [13], blunting of the villi and disorganization of crypts (location of stem cells) were the primary events associated with severe mucositis. The jejunum is the maximal site of injury induced by chemotherapy and the impact becomes less pronounced in the more distal regions of the small intestine [13]. This was demonstrated in the current study in which less injury was observed in terms of MPO activity (neutrophil infiltration), villus height and mucosal thickness at the distal end of the small intestine

The bioavailability of PCs in the gut system has been well documented in other studies [24]. The unique polymerized structure of PCs inhibits absorption across the small intestine, as they adhere to the gut mucosa [25]. Tsang *et al.* [26] detected larger forms of PCs in the small intestine of rats up to 12 h after ingestion. Thus, an accumulation of relatively high PC concentrations can occur in the gut lumen to protect the intestinal barrier. In the current study, higher doses of GSE (1000 mg/kg) were effective at maintaining crypt depth and mucosal thickness in the jejunal region, with most values approaching the values of healthy controls. Furthermore, the current study also showed improvement of fecal output (less severe diarrhoea) in chemotherapy-treated rats receiving the higher dose of GSE (600 mg/kg and 1000 mg/kg), suggesting reduced disruption of the mucosal lining of the small intestine.

Although GSE in the current study was more effective in the jejunum, the site of major intestinal injury, bioactivity was reduced in the distal small intestine. This may have been due to degraded bioactive components reaching the distal region of the small intestine [27], [28]. The cleavage, absorption and metabolism of GSE is important to identify the fate of bioactive compounds in GSE. In future studies it will be necessary to identify the form, size and bioactivity of procyanidins from GSE responsible for promoting intestinal health using *in vitro* and *in vivo* models of intestinal absorption. Additionally, future studies could examine protection of GSE, possibly by microencapsulation, or via suppository application, to better target GSE and improve its bioavailability in the more distal regions of the bowel. Due to the complexity of GSE content, it would be difficult to determine which factors are responsible for the observed bioactivity. For this reason, GSE, rather than alternative protein source such as bovine serum was used as its own control. Administration of GSE on normal animals allowed more precise comparison with GSE-treated rats receiving 5-FU chemotherapy.

Interest in GSE has been primarily due to its high antioxidant content. GSE is a more potent radical scavenger than other known anti-oxidants such as vitamin C and E [29]. In the present study, the partial reduction in acute inflammation by GSE, as indicated by the decrease of MPO activity, and reduction in lymphocyte infiltration recorded by the disease severity score analysis, could strengthen the potential role of GSE as a potent anti-oxidant and anti-inflammatory agent. A number of studies have described GSE as an anti-inflammatory agent. For example, GSE has been reported to reduce the expression of pro-inflammatory cytokines (TNF-α and IL-6) in mesenteric lymph nodes [30], rat plasma [31] and carrageenan-induced paw edema in rats [32]. The reduction of these activities may represent a consequence of GSE and its ability to prevent NF-κB activation and subsequently reduce the activation of nitric oxide and pro-inflammatory cytokines. Thus, inhibition of NF-κB activation may have been a possible mechanism by which GSE reduced mucosal injury and hence mucositis severity, in the current study. Other biomarkers such as inflammatory cytokines in tissue and blood could be measured in future studies to quantify GSE effects on the systemic and mucosal immune system.

PC rich food has been reported to be both beneficial and detrimental to human health due to its ability to interact with proteins (enzymes, toxins, hormones) [10], [33]. The current study provides important information on the safety of GSE usage. Oral administration of GSE (400 mg/kg, 600 mg/kg and 1000 mg/kg) for nine days did not induce any deleterious side-effects in healthy animals. The increased of stomach weight in GSE treated rats might be due to indigestible of GSE PCs deposited in the stomach. GSE did not impact negatively on daily metabolic parameters, nor induce any side-effects in the small intestine. Moreover, the sucrose breath test indicated that GSE did not affect small intestinal brush border enzyme activity. These data concur with other studies [34] in which rats ingesting up to 2 g/kg of GSE showed no abnormal metabolic findings or toxicological effects. In the current study, GSE (1000 mg/kg) significantly increased stomach weight in healthy rats. This finding has not been reported previously [35], but could be a reflection of differing rat strains between studies. Thus, histological analyses on rat stomach should be conducted in future studies.

The promising effects of GSE in the mucositis rat model provided the impetus to further investigate its potential impact on the effectiveness of chemotherapy against transformed colonocytes. Recently, various strategies have been developed to counter the development of mucositis (reduced gastrointestinal toxicity) or to enhance the chemotherapeutic activity of 5-FU. To date, a number of strategies to enhance 5-FU efficacy on colon cancer have been investigated although none are clinically available. These include omega 3-fatty acid [36], chloroquine [37], violecin [38] and ginseng [39] which have been reported to effectively improve 5-FU efficiency at killing cancer cells *in vitro* compared to the chemotherapy agent acting independently. In the current study, the effects of GSE and 5-FU on Caco-2 cell viability were examined at 24 h and 48 h. This was because 5-FU (100 uM) significantly reduced cell viability to 70–85%, reflecting gastrointestinal toxicity in cancer patients. Moreover, longer time exposure (24–48 h) of cancer cells to both GSE and 5-FU resulted in a further reduction in cell viability. However, the differences between treatments remained relatively unchanged even up to 72 h (data not shown). Importantly, GSE acted synergistically with 5-FU to inhibit Caco-2 cell proliferation.

GSE may act as a potent chemotherapeutic agent as it has been demonstrated to exert selective cytotoxicity against tumour cells compared to normal cells [40], [41]. It has been suggested that GSE induces growth inhibition in cancer cells via induction of cell cycle arrest which eventually leads to the induction of caspase-dependent apoptosis [8] and disruption of the mitochondrial membrane [42]. Moreover, the current study demonstrated that GSE alone at higher concentration, tended to induce greater growth inhibitory effects on Caco-2 cells compared to 5-FU alone. Thus, the current data support GSE as a promising anti-neoplastic adjunct to cancer treatment. Future *in vitro* studies including invasion, proliferation and growth analysis should be performed to identify the phenotypic changes of colon cancer cells after GSE treatment.

Although the current *in vivo* study revealed that GSE only minimally improved parameters of intestinal mucositis (disease severity score), future studies could examine the efficacy of higher doses of GSE or alternatively more highly purified PC compounds. Chemotherapy is likely to progress to a chronic condition. Future studies should investigate GSE effects in rat models over protracted periods of several weeks and months. The promising findings of GSE in the *in vitro* model also support further studies into the identification of bioactive components of GSE responsible for these effects. The present study was conducted using female Dark Agouti rats which can be manipulated to develop breast cancer [43]. Such studies would facilitate further investigations into GSE and its potential to modify tumour growth. The colon cancer cell line was selected on the basis that it could be translated later to an animal model of colon cancer. The current work represents the synergistic effect of GSE and 5-FU at partially preventing mucositis, whilst reducing Caco-2 cells viability. This will allow us to further investigate GSE for its potential to modify tumour growth with 5-FU by determining its effects on tumour growth such as that induced by azoxymethane in rat model [44].

In conclusion, the current investigation provides the first evidence for GSE to reduce the severity of intestinal mucositis in a dose-responsive manner while enhancing the impact of 5-FU chemotherapy on colon cancer cells. Dietary GSE could be a promising adjunctive approach for combating intestinal mucositis while concurrently potentiating the impact of conventional chemotherapy for colon cancer.
